# Regulation of DNA replication initiation by ParA is independent of *parS* location in *Bacillus subtilis*

**DOI:** 10.1099/mic.0.001259

**Published:** 2022-10-01

**Authors:** Alan Koh, Henrik Strahl, Heath Murray

**Affiliations:** Centre for Bacterial Cell Biology, Institute for Cell and Molecular Biosciences, Newcastle University, Newcastle Upon Tyne, NE2 4AX, UK

**Keywords:** DNA replication initiation, chromosome segregation, ParB, ParA, *parS*, *oriC*, DnaA, *Bacillus subtilis*

## Abstract

Replication and segregation of the genetic information is necessary for a cell to proliferate. In *Bacillus subtilis*, the Par system (ParA/Soj, ParB/Spo0J and *parS*) is required for segregation of the chromosome origin (*oriC*) region and for proper control of DNA replication initiation. ParB binds *parS* sites clustered near the origin of replication and assembles into sliding clamps that interact with ParA to drive origin segregation through a diffusion-ratchet mechanism. As part of this dynamic process, ParB stimulates ParA ATPase activity to trigger its switch from an ATP-bound dimer to an ADP-bound monomer. In addition to its conserved role in DNA segregation, ParA is also a regulator of the master DNA replication initiation protein DnaA. We hypothesized that in *B. subtilis* the location of the Par system proximal to *oriC* would be necessary for ParA to properly regulate DnaA. To test this model, we constructed a range of genetically modified strains with altered numbers and locations of *parS* sites, many of which perturbed chromosome origin segregation as expected. Contrary to our hypothesis, the results show that regulation of DNA replication initiation by ParA is maintained when a *parS* site is separated from *oriC*. Because a single *parS* site is sufficient for proper control of ParA, the results are consistent with a model where ParA is efficiently regulated by ParB sliding clamps following loading at *parS*.

## Introduction

The ability for cells to coordinate DNA replication with chromosome segregation is critical for execution of the cell cycle. In most prokaryotes the processes of DNA replication and segregation overlap, with newly replicated DNA regions immediately separated from one another. Primary bacterial chromosomes generally harbour a single origin of replication (*oriC*). Binding of the master initiator protein DnaA to *oriC* promotes specific DNA unwinding and allows the loading of the replication machinery [1]. Following duplication of *oriC*, the organization and movement of this chromosomal region is critical for subsequent bulk chromosome segregation [2].

Bacterial DNA partitioning (*par*) genes were first identified as elements required for accurate segregation of low copy-number plasmids into daughter cells. The *par* locus consists of three elements, two *trans*-acting proteins (ParA and ParB) and a *cis*-acting binding sequence (*parS*). All three elements are essential for efficient inheritance of plasmid DNA into daughter cells [3–5]. Homologous partitioning genes are harboured within the majority of bacterial chromosomes and bioinformatic analysis revealed that *parS* sites generally cluster near *oriC*, suggesting that cellular Par systems are devoted to regulating processes involving the origin region of the bacterial genome [6].

ParB, known as Spo0J in *B. subtilis* because the *spo0J* mutation confers a classic sporulation defect, is a dimeric CTP-dependent DNA sliding clamp [7–14]. ParB^CTP^ binds specifically to a *parS* site, which stimulates the ParB N-terminal nucleotide binding domain to dimerize, causing ParB to adopt an annular structure that can slide along the DNA [9, 15]. In *B. subtilis*, a *parB* null mutant both overinitiates DNA replication and produces anucleate cells, indicating that it plays roles in both DNA synthesis and chromosome segregation [8, 16–19].

ParA, known as Soj in *B. subtilis* (suppressor of *spo0J*), is a dynamic ATPase [8, 20] that changes oligomeric state and activity depending upon the bound adenine nucleotide [21]. It has been proposed that ParA^ADP^ is a monomer which can interact directly with DnaA to inhibit the initiation of DNA replication [16, 17, 22]. ParA^ATP^ forms a homodimer that binds DNA in a sequence non-specific manner and promotes DnaA-dependent DNA replication initiation [16, 17, 22]. ParB stimulates the ATPase activity of ParA to generate ParA^ADP^ monomers, thus triggering its switch from an activator to an inhibitor of DnaA [16, 17].

As in other Par systems, ParA plays a role in chromosome segregation, specifically facilitating bidirectional origin movement following DNA replication initiation [2]. Current evidence indicates that ParA^ATP^ binds to DNA in a non-specific manner, forming a gradient over the chromosome that attracts ParB: *parS* nucleoprotein complexes [23–26]. The interaction of ParB with dimeric ParA activates its ATPase activity, converting it to ParA^ADP^ monomers that cannot bind DNA and thus diffuse away from the ParB nucleoprotein complexes. The dispersion of ParA^ADP^ from the DNA creates a path of ParA^ATP^ dimers on the chromosome that can attract ParB, thereby working as a Brownian ratchet to couple the ATPase-dependent cycling of ParA to the directed movement of ParB:*parS* nucleoprotein complexes [23–26].

In addition to the Par system, the *B. subtilis* structural maintenance of chromosome (SMC) condensin complex is recruited to the *oriC* region by ParB:*parS* nucleoprotein complexes where it organizes the chromosome origin macrodomain and facilitates separation of newly replicated origins, particularly during rapid cell growth [27–32]. In nutrient rich media, null mutants in any of the genes encoding the SMC complex leads to growth inhibition, anucleate cell formation, and aberrant nucleoid structure [27–32].

**Figure 1:**
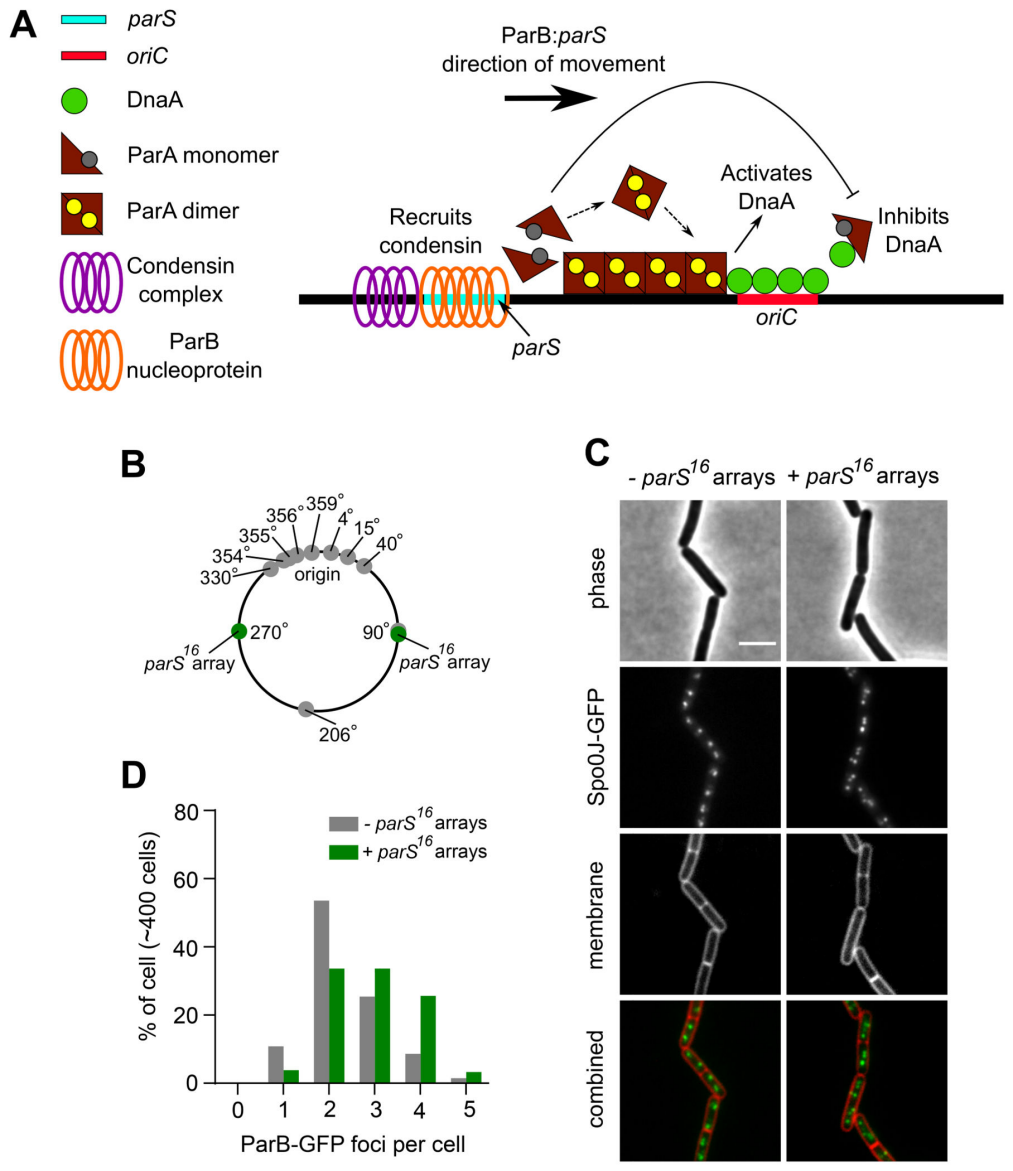
Chromosomal *parS^16^* arrays redistribute ParB. **(A)** Schematic of a two-dimensional ParB nucleoprotein complex along a *parS* with the left section showing ParB interacting with condensin for chromosome organization and segregation. The right section showing ParA spreading across the DNA in a gradient, with ParB regulating the activity cycle of ParA. Regulation of initiation of DNA replication depends on the oligomeric state of ParA. In addition, the diffusion ratchet manner of ParB-ParA interactions allow the segregation of replicated sister origins. For simplicity, ATP and ADP is shown as a yellow and grey circle respectively. **(B)** Schematic diagram showing the location of *B. subtilis* endogenous chromosomal *parS* (grey circles) and the *parS^16^* arrays (green circles). **(C)** ParB-GFP localization in a wild-type strain and in a strain harbouring *parS^16^* arrays. Phase contrast (top panel), ParB-GFP (middle panel) and outer membrane dye FM-595 (bottom panel). **(D)** The number of ParB-GFP foci per cell increased in the presence of *parS^16^* arrays. Scale bar, 3 μm.

It has been established that ParB:*parS* complexes must be located near *oriC* to promote origin region segregation through the ParA and condensin systems [27, 29, 30]. We hypothesized that origin proximal localization of ParB:*parS* nucleoprotein complexes might also be required for proper regulation of DNA replication initiation by ParA (via DnaA at *oriC*). In this model the Par system would act to coordinate the processes of DNA segregation and replication (Fig. 1A).

## Results

### Additional *parS* sites alter ParB localization and disrupt chromosome origin segregation

**Figure 2:**
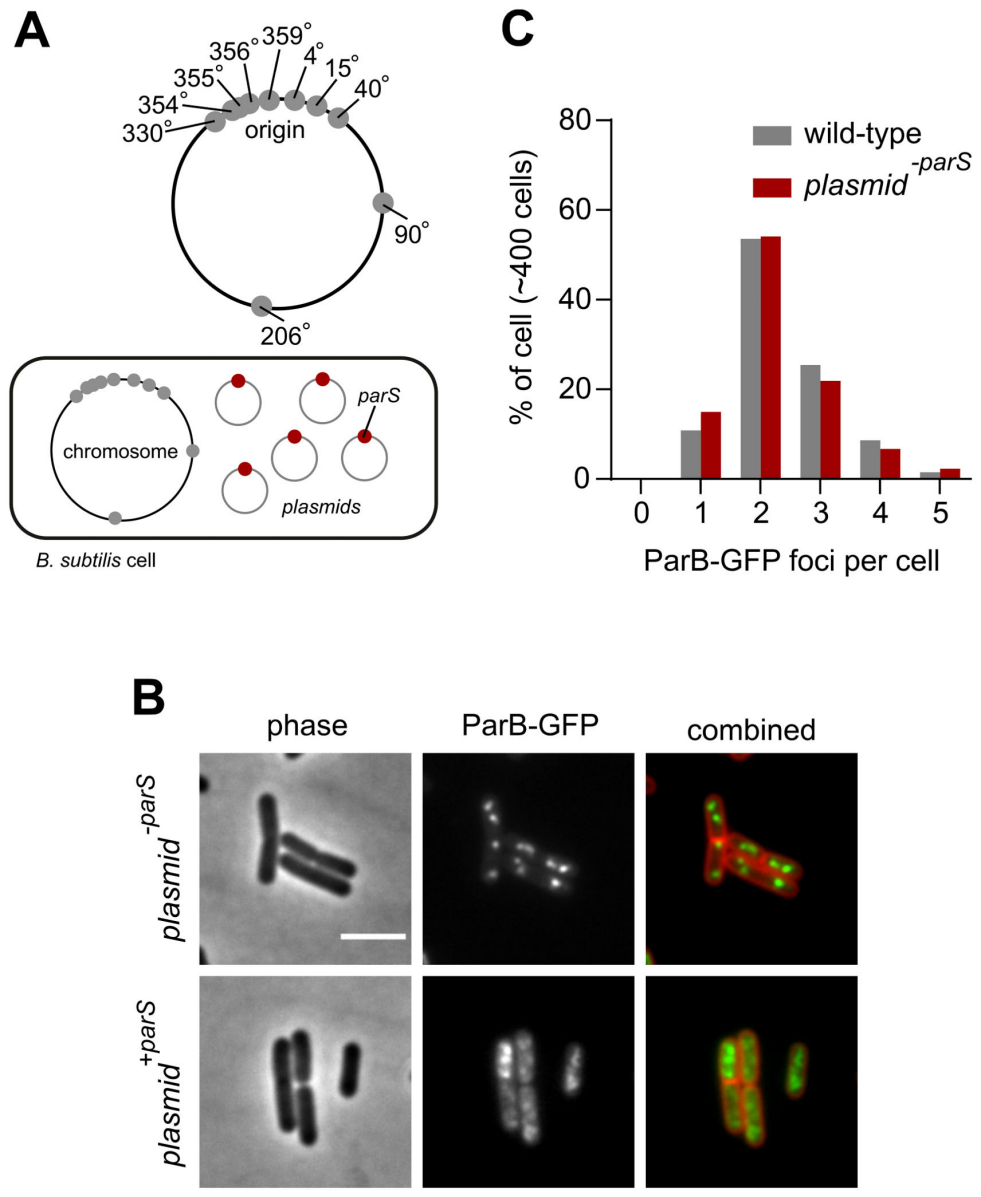
Extrachromosomal *parS* redistribute ParB. **(A)** Schematic diagram showing the location of *B. subtilis* endogenous chromosomal *parS* (grey circles) and plasmid containing a single *parS* (red circles). **(B)** ParB-GFP localization in a wild-type strain harbouring plasmids that contain either a single or no parS. Phase contrast (left panel), ParB-GFP (middle panel) and membrane dye FM 5-95 combined with ParB-GFP (right panel). **(C)** The number of ParB-GFP foci per cell remains unchanged in the presence of the empty vector. Scale bar, 3 μm.

To investigate whether the localization of ParB:*parS* nucleoprotein complexes proximal to *oriC* is required for the proper regulation of DNA replication initiation, strains were constructed to redistribute ParB away from the endogenous *parS* sites located within the chromosome origin region. In one approach, two *parS*^16^ arrays (16 tandem repeats of the consensus *parS* sequence 5´-TGTTCCACGTGAAACA-3´ [19]) were integrated into the chromosome, one set in each chromosome arm (Fig. 1B). In a second approach, a single consensus *parS* site was engineered into a high copy-number plasmid (Fig. 2A). Note that in both strains the endogenous *parS* sites are unaltered.

To determine the effect of additional *parS* sites on ParB activity, the endogenous *parB* gene was replaced by a functional *parB-gfp* fusion and epifluorescene microscopy was used to determine the localization of ParB-GFP. Under the slow growth conditions used in these experiments, the majority of wild-type cells contained a pair of ParB-GFP foci located towards the cell poles, which is characteristic of *oriC* localization in *B. subtilis* (Figs. 1C-D) [33, 34]. A control plasmid lacking *parS* did not alter this pattern (Figs. 2B-C). In contrast, both strains harbouring additional *parS* sites displayed marked differences. The chromosomal *parS* arrays resulted in an increase in the number of discreet ParB-GFP foci per cell (Figs. 1C-D), while the extrachromosomal *parS* site appeared to disturb ParB-GFP foci formation (Fig. 2B). Analysis of both ParB-GFP fluorescence in single cells and ParB levels using immunoblotting indicate that ParB expression is unchanged in strains with additional *parS* sites (Fig. S1). Together, the localization results suggest that additional *parS* sites recruit a population of ParB molecules away from *oriC*.

**Figure 3:**
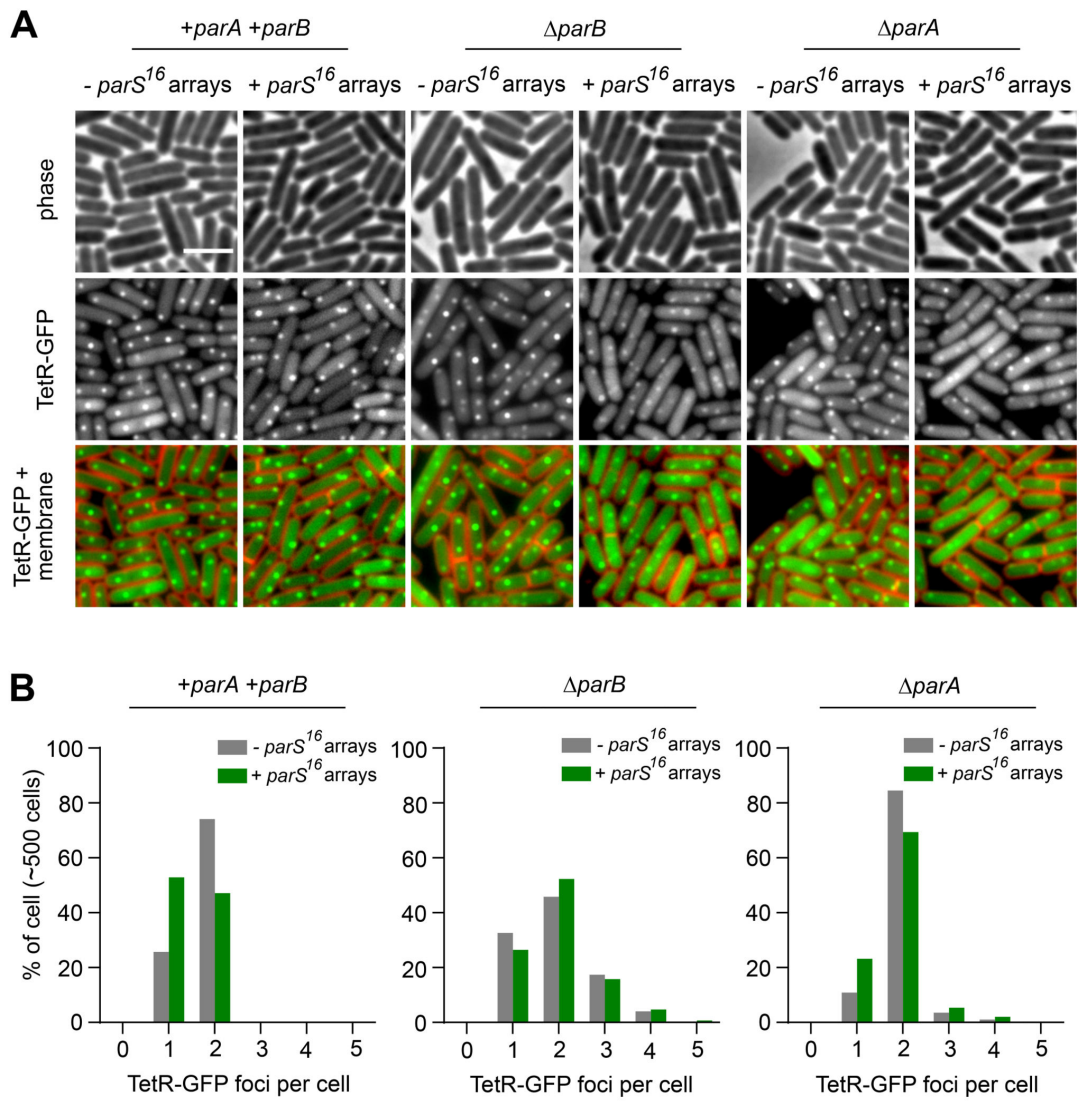
Chromosomal *parS^16^* arrays redistribute ParB to disrupt chromosome origin segregation. **(A)** Origin localization in a wild-type strain and in a strain harbouring *parS^16^* arrays. The *oriC* region was labelled using an array of *tetO* operators bound by TetR-GFP. Phase contrast (top panel), TetR-GFP (middle panel) and membrane dye FM 5-95 combined with TetR-GFP (bottom panel). **(B)** ParB dependent decrease in the number of origin per cell in the presence of *parS^16^* arrays. Scale bar, 3μm.

**Figure 4:**
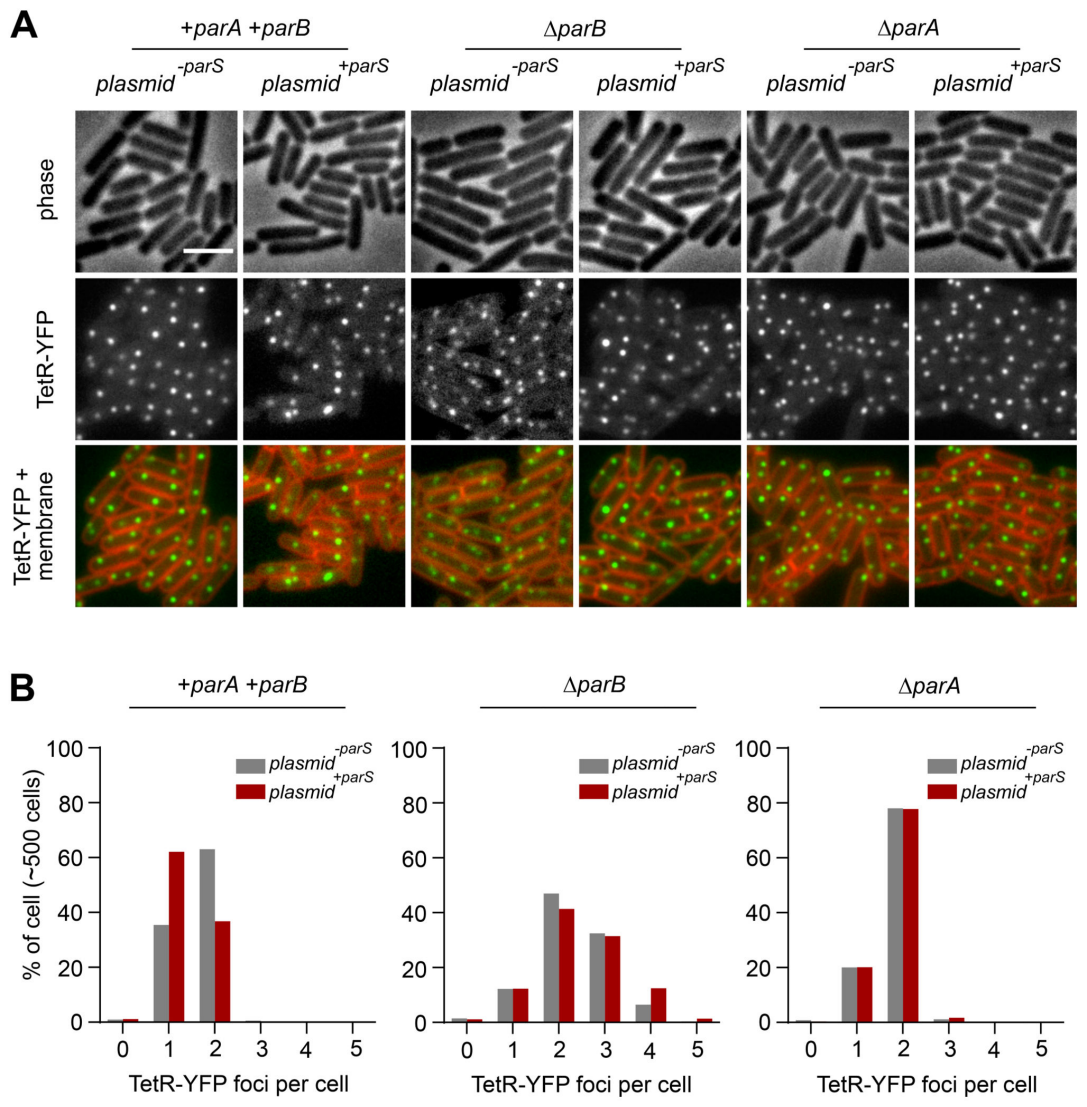
Extrachromosomal *parS* redistribute ParB to disrupt chromosome origin segregation. **(A)** Origin localization in a wild-type strain and in a strain harbouring *plasmid ^+parS^*. Origin region was labelled using an array of *tetO* operators bound by TetR-YFP. Phase contrast (top panel), TetR-YFP (middle panel) and membrane dye FM 5-95 combined with TetR-YFP (bottom panel). **(B)** ParB dependent decrease in the number of origin per cell in the presence of *plasmid ^+parS^*. Scale bar, 3μm.

To investigate the consequence of ParB redistribution on *oriC* segregation, the origin region was visualised using a fluorescent repressor-operator system (*tetO* operators bound by TetR-GFP or TetR-YFP). As observed with ParB-GFP, wild-type cells contained two TetR-FP foci located near the cell poles (Figs. 3A-B, 4A-B). In contrast, when additional *parS* sites were present, there was an increase in the number of cells with a single fluorescent focus and a corresponding decrease in the number of cells with two foci, consistent with a defect in *oriC* separation (Figs. 3A-B, 4A-B). In a Δ*parB* mutant the number of origins per cell was increased independent of *parS* copy-number, indicating that the origin segregation defect is ParB-dependent and consistent with previous results showing that DNA replication initiation is stimulated in the absence of ParB (Figs. 3A-B, 4A-B) [16, 17, 22]. Interestingly, the origin separation defects caused by additional *parS* sites were diminished in a Δ*parA* mutant (Fig. 3A-B, 4A-B). This observation suggests that ParA exerts a negative effect on *oriC* segregation, possibly by perturbing the interaction between ParB and condensin near the chromosome origin [27, 28].

### Control of DNA replication initiation by ParA is not affected by increasing the number of *parS* sites

**Figure 5:**
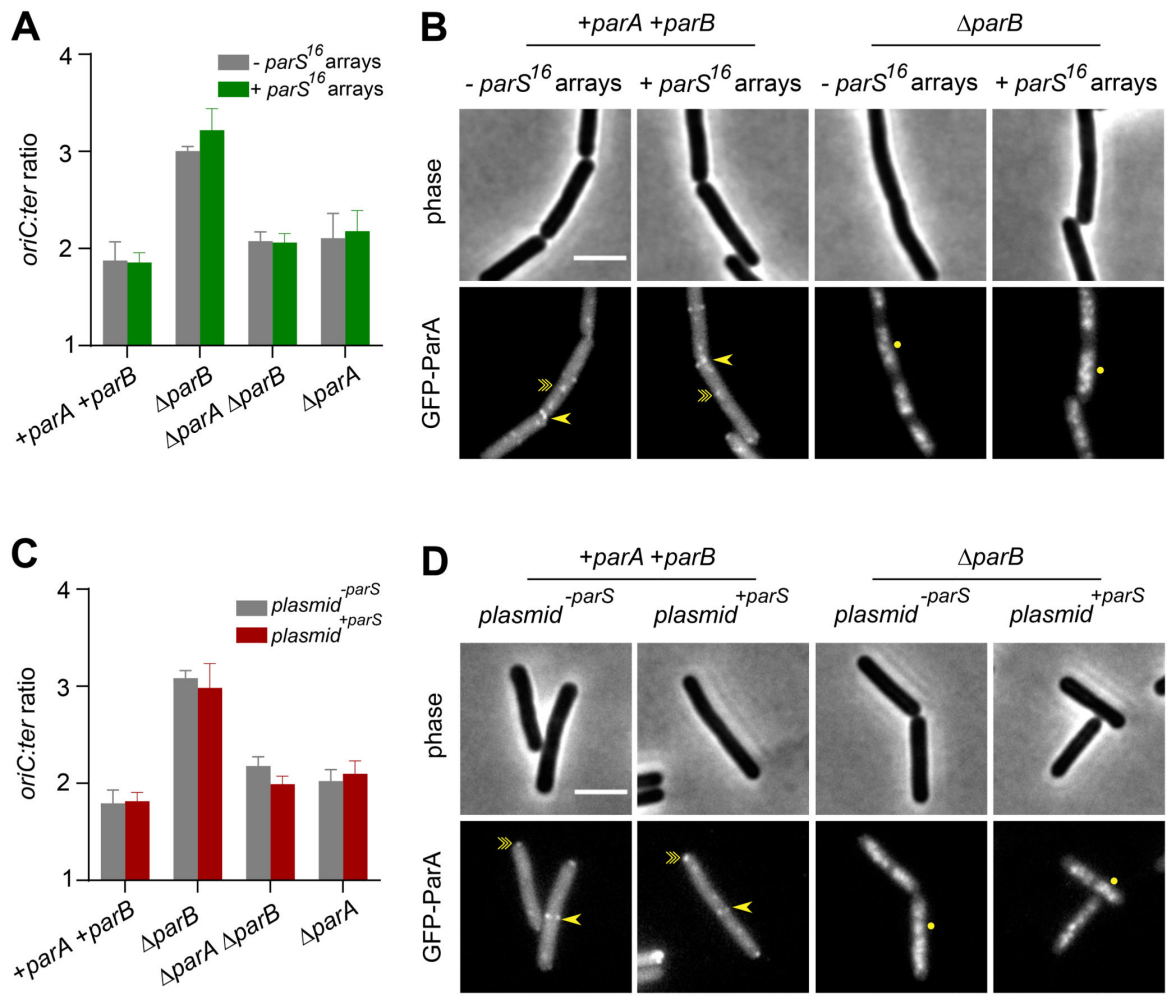
ParA activities are unperturbed in the presence of additional *parS*. **(A)** The frequency of DNA initiation was not affected by chromosomal *parS^16^* arrays. The *oriC*:*ter* ratio of each strain was determined using quantitative PCR. **(B)** GFP-ParA localization in strains containing chromosomal *parS^16^* arrays. A single-point arrow (->) denotes localization at a septum, a double-point arrow (-≫) indicates localization as a focus and a circle (●) denotes nucleoid binding. **(C)** The frequency of DNA initiation was not affected by extrachromosomal *parS*. The *oriC*:*ter* ratio of each strain was determined using quantitative PCR. **(D)** GFP-ParA localization in strains containing *plasmid ^+parS^*. A single-point arrow (->) denotes localization at a septum, a double-point arrow (-≫) indicates localization as a focus and a circle (●) denotes nucleoid binding. Scale bar, 3μm.

After establishing that additional *parS* sites redistribute ParB away from *oriC* and impair origin segregation, we next measured the rate of DNA replication initiation. Marker frequency analysis did not detect any significant change in the *ori:ter* ratio when additional *parS* sites were present, indicating that regulation of DnaA by ParA was retained (Figs. 5A, 5C). These results also suggest that the decrease in the number of TetR-FP foci per cell caused by additional *parS* sites was due to a defect in *oriC* segregation (Figs. 3A-B, 4A-B), rather than a decrease in the rate of DNA replication initiation. A significant ParA-dependent increase in the rate of replication initiation was observed when *parB* was deleted, showing that ParA remains competent to regulate DnaA in the presence of additional *parS* sites (Figs. 5A, 5C).

To confirm that ParA was being properly regulated in strains containing additional *parS* sites, the localization of GFP-ParA was determined. The *parA* gene was fused to *gfp* under the control of its native promoter and GFP-ParA was visualized using epifluorescence microscopy. Previous work has shown that in a wild-type strain GFP-ParA forms both DnaA-dependent foci at *oriC* and localizes to septa, while in a Δ*parB* mutant GFP-ParA colocalizes with the nucleoid (Figs. 5B, 5D) [16, 17, 21, 35, 36]. The pattern of GFP-ParA localization in strains containing additional *parS* sites was similar to the parent strains (Figs. 5B, 5D). Taken together with the marker frequency analysis, the results indicate that regulation of ParA by ParB, and thereby control of DNA replication initiation by ParA, is not affected by additional *parS* sites that titrate ParB away from *oriC*. Moreover, these experiments show that regulation of DnaA by ParA is retained under conditions when chromosome origin separation is disrupted, indicating that ParA does not mediate a causal relationship between DNA segregation and replication in *B. subtilis*.

### A single *parS* site is necessary and sufficient to control ParA activity

**Figure 6:**
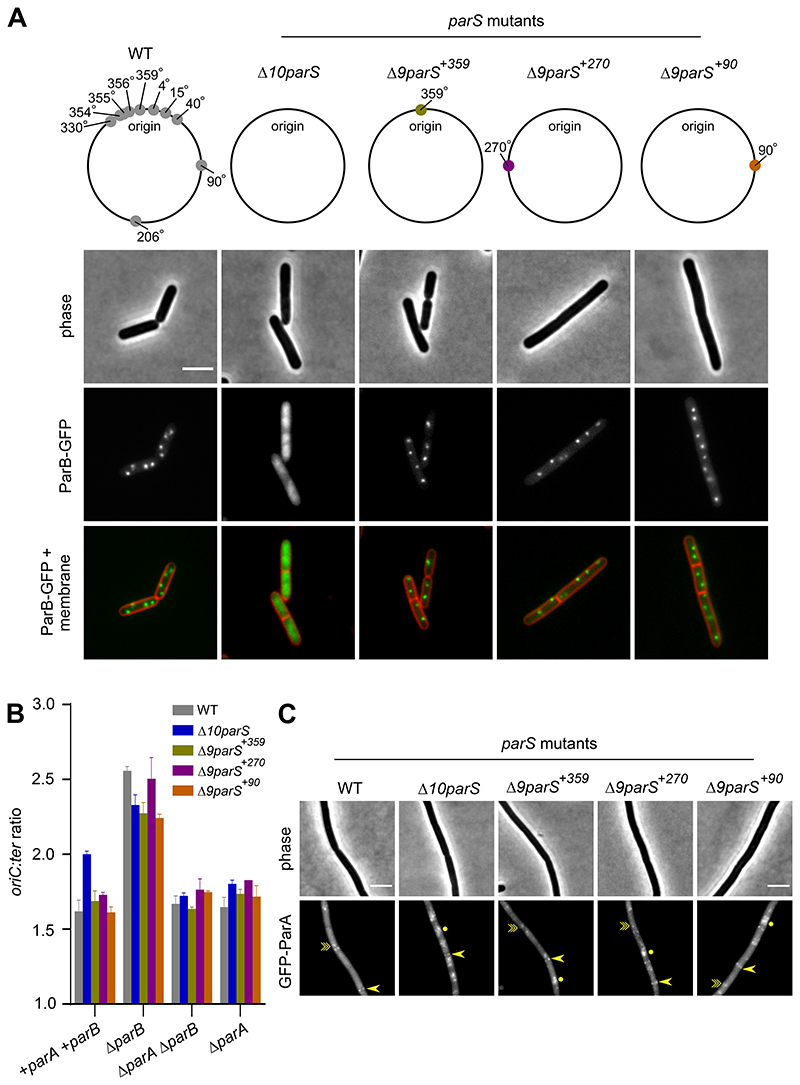
A single chromosomal *parS* is necessary and sufficient to regulate ParA activity. **(A)** ParB-GFP localization in strains that differ in the location of *parS*. Phase contrast (top panel), ParB-GFP (middle panel) and membrane dye FM 5-95 combined with ParB-GFP (bottom panel). Schematic diagram showing the location of *B. subtilis* endogenous chromosomal *parS* (grey circles), *parS* at 359° (green circle), *parS* at 270° (purple circle) and *parS* at 90° (orange circle). **(B)** The frequency of DNA initiation was restored by ParB sliding clamps loading at a single *parS*. The *oriC-ter* ratio of each strain was determined using quantitative PCR. **(C)** GFP-ParA localization in strains that differ in the location of *parS*. A single-point arrow (->) denotes localization at a septum, a double-point arrow (-≫) indicates localization as a focus and a circle (●) denotes nucleoid binding. Scale bar, 3 μm.

Although recruitment of ParB to additional *parS* sites appears sufficient to impair chromosome origin separation, in these strains the endogenous *parS* sites flanking *oriC* remained intact. Therefore, we wondered whether residual ParB binding to these *oriC*-proximal *parS* sites was responsible for regulating ParA. To address this question a strain was constructed with all ten *parS* sites deleted (Δ10*parS*). In the Δ10*parS* mutant, ParB-GFP foci formation was lost, confirming that no *parS* sites are present in the strain (Fig. 6A). Marker frequency analysis showed that the rate of DNA replication initiation was increased in the Δ10*parS* mutant, and deletion of *parA* from the Δ10*parS* strain confirmed that this effect was ParA-dependent (Fig. 6B). Consistent with the interpretation that ParA activity was altered in the absence of *parS* sites, the localization of GFP-ParA was shifted towards a nucleoid-bound state in the Δ10*parS* strain, a similar pattern to what is observed in a Δ*parB* mutant (Figs. 5B, 5D, 6C).

To investigate whether a single *parS* site is sufficient to regulate ParA, the native *parS^359^* site was re-introduced near *oriC* (Δ9*parS^+359^*). ParB-GFP foci were restored in the Δ9*parS^+359^* strain, suggesting that ParB sliding clamps were being loaded at *parS* (Fig. 6A). Marker frequency analysis showed that the rate of DNA replication initiation in the Δ9*parS^+359^* strain was comparable to wild-type (Fig. 6B). Deletion of *parB* in the Δ9*parS^+359^* strain resulted in a ParA-dependent increase in the rate of DNA replication initiation (Fig. 6B), indicating that ParA is functional and being properly controlled by the single *parS* site. Consistent with the interpretation that ParA regulation remains intact in Δ9*parS^+359^* strain, GFP-ParA localization was generally similar to its pattern in the wild-type strain (although there was an increase in the number of patchy fluorescent signals, likely representing interactions with the chromosome) (Fig. 6C). Together, these results indicate that a single *oriC*-proximal *parS* site is necessary and sufficient to regulate ParA.

To test whether the *parS* site needs to be located near *oriC* for ParA to regulate DNA replication initiation, a single *parS* site was reintroduced into the Δ10*parS* strain far from the origin region. We created two strains, with the *parS* site located in either the left or the right chromosome arm (Δ9*parS^+90^* or Δ9*parS^+270^*). In both strains ParB-GFP formed foci within the cell, confirming that the reintroduced *parS* site was functional (Fig. 6A). As expected, locating a *parS* site away from *oriC* leads to cells displaying aberrant chromosome and cell morphologies (Fig. S2) [27–32]. In addition, examination of *oriC* number per cell in the Δ9*parS^+90^* strain indicated a ParA-dependent origin separation defect (Figs. S3A-B). Critically however, marker frequency analysis showed that the rate of DNA replication initiation in both the Δ9*parS^+90^* and Δ9*parS^+270^* strains was similar to wild-type (Fig. 6B). Control of DNA replication initiation in the Δ9*parS* strains remained intact, as ParA-dependent over-initiation of DNA replication was observed when *parB* was deleted (Fig. 6B). In agreement with these observations, localization of GFP-ParA in either the Δ9*parS^+90^* or Δ9*parS^+270^* strain resembled the wild-type pattern, with the protein forming faint cytoplasmic foci and binding to septa (although like the Δ9*parS^+359^* strain an increase in the number of patchy fluorescent signals was observed, likely representing chromosome binding) (Fig. 6C). Taken together, the results indicate that a *parS* site does not need to be located near *oriC* for it to dictate ParA activity at the chromosome origin.

## Discussion

Par systems promote chromosome origin separation because *parS* sites are located near *oriC*. We hypothesized that *parS* positioning near *oriC* would also be important for the Par system to regulate the frequency of DNA replication initiation. Contrary to this proposed model, the data indicates that only a single *parS* site, independent of its location, is necessary and sufficient to maintain proper regulation of ParA. Moreover, control of DnaA activity by ParA was maintained even when the process of *oriC* separation was severely disrupted, indicating that ParA does not provide a functional link connecting origin segregation with DNA replication initiation in *B. subtilis*.

During the majority of the *B. subtilis* cell cycle, ParB acts to stimulate the ATPase activity of ParA and thereby promote monomer formation, which in turn inhibits DnaA activity [16, 17]. Because a *parS* site is required to properly regulate ParA, taken together with recent work describing the ParB:*parS* reaction cycle [9–14], the results suggest that ParB is most efficient at regulating ParA as a sliding clamp encircling DNA.

It remains unclear when or where ParA accumulates as a dimer to stimulate DnaA activity in *B. subtilis*. One possibility is that the rate of ParB clamp loading at *parS* could fluctuate, either during the cell cycle or between growth phases. We considered whether passage of the replisome through the origin region might displace ParB from the chromosome. Stimulation of ParA ATPase activity by ParB is 40-fold less efficient when the proteins are in solution without DNA [22]. However, in this scenario a new round of DNA replication would have just recently been initiated. Although this would appear to be an undesirable time to activate DnaA, it is important to note that DnaA is also a cell cycle regulated transcription factor [37]. For example, DnaA positively activates transcription of the developmental checkpoint gene *sda* to coordinate DNA synthesis with initiation of endosporulation, and dispersal of ParB from the chromosome might act as a signal to synchronize the DNA replication initiation with Sda expression [38]. Another possibility is that the ParB clamp could be modified to alter the interaction with ParA, either directly through interactions with proteins such as condensin or indirectly through changes in chromosome conformation [29, 30, 32].

Finally, current understanding of the opposing effects ParA has on DnaA is based mainly on genetic analysis of *parA* mutants [16, 17, 22]. It will be critical for future studies to analyze the dynamic behaviour of ParA, ParB, and DnaA within the context of a natural cell cycle to determine when and where they functionally interact.

## Experimental Procedures

### Strains and growth conditions

All strains used in this study are listed in Supplementary Table 1, and were routinely maintained on nutrient agar (NA) (Oxoid) with supplements. Selective media contained chloramphenicol (5 μg/ml), erythromycin (1 μg/ml), kanamycin (2 μg/ml), zeomycin (10 μg/ml), tetracycline (10 μg/ml), spectinomycin (50 μg/ml), phleomycin (1 μg/ml), ampicillin (100 μg/ml). For experiments in *B. subtilis*, overnight cells were grown in defined minimal medium base (Spizizen minimal salts supplemented with Fe-NH_4_-citrate (1 μg/ml), MgSO_4_ (6 mM), CaCl_2_ (100 μM), MnSO_4_ (130 μM), ZnCl_2_ (1 μM), thiamine (2 μM), casein hydrolysate (200 μg/ml), tryptophan (20 μg/ml) and succinate (2.0%) as carbon sources. Subsequent fresh media used for cell dilution were as mentioned above with either succinate (2.0%) or glucose (2.0%) as carbon source but without casein hydrolysate.

### DNA manipulation and strain constructions

All *B. subtilis* transformations were carried out via a two-step starvation protocol as previously described (Anagnostopoulos and Spizizen 1961 and Hamoen 2002). DNA for transformation was provided as either genomic DNA from a *B. subtilis* strain or linearized plasmid DNA containing the desired genetic construct. Details of individual strain constructions and plasmids genotype are listed in Supplementary Table 1 and 2 respectively. Oligonucleotides used in plasmid construction are listed in Supplementary Table 3. All plasmids and strains were verified by sequencing.

### Marker frequency analysis

For measurement of DNA replication, starter cultures were grown overnight at 37˚C in SMM based medium, before resuspension in fresh medium containing succinate (2.0%) as the carbon source the following day. Cultures were then allowed to undergo at least three doubling to early exponential phase at 37˚C. Sodium azide (0.5%; Sigma) was added to exponentially growing cells to prevent further metabolism. Chromosomal DNA was isolated using a DNeasy Blood and Tissue Kit (Qiagen). Either Rotor-Gene SYBR Green (Qiagen) or GoTaq (Promega) qPCR mix was used for PCR reactions. Q-PCR was performed in a Rotor-Gene Q Instrument (Qiagen). For quantification of the origin, the intergenic region between *dnaA* and *dnaN* was amplified using primers 5’-GATCAATCGGGGAAAGTGTG-3’ and 5’-GTAGGGCCTGTGGATTTGTG-3’. For quantification of the terminus, the region downstream of *yocG* was amplified using primers 5’-TCCATATCCTCGCTCCTACG-3’ and 5’-ATTCTGCTGATGTGCAATGG-3’. By use of crossing points (C_T_) and PCR efficiency a relative quantification analysis (∆∆C_T_) was performed using Rotor-Gene Software version 2.0.2 (Qiagen) to determine the *ori/ter* ratio of each sample. These results were normalized to the *ori/ter* ratio of a DNA sample from *B. subtilis* spores which only contain one chromosome and thus have an *ori/ter* ratio of 1. At least two biological repeats were performed for each experiments with error bars indicating the standard deviation of at least two technical replicates from one experimental set.

### Epifluorescence microscopy

For fluorescent microscopy analysis, cells were grown at 37˚C in accordance with the marker frequency analysis using glucose (2.0%) as the carbon source for cell resuspension. Individual cell membrane were stained with 0.4 μg/ml FM5-95 (N-(3-Trimethylammoniumpropyl)-4-(6-(4(Diethylamino)phenyl)hexatrienyl) Pyridinium Dibromide) from ThermoFisher Scientific. Cells were immobilized onto 1.2% agarose in 0.25x SMM base medium spread thinly onto microscope slides. Microscopy was carried out with Nikon Eclipse Ti equipped with Nikon DM 100x/1.40 Oil Ph3 objective, Photometrics CoolSnap HQ^2^ cooled CCD camera and light source was Lambda LS (Sutter Instrument). Metamorph V.7.7 were used to acquire images. Cells were quantified using Fiji (Schindelin *et al*., 2012). At least three biological repeats were performed for each experiment.

### Fluorescent intensity measurement

The average fluorescent intensity of ParB-GFP was measured as described [39]. Intensity was generated by averaging 11 pixels (0.72 μm) aligned perpendicular to the cell length axis, and by measuring along the length axis of the cell. Cells were quantified using Fiji [40].

### Western blot analysis

For proteins visualization, cells were grown in accordance with general fluorescent microscopy analysis. Proteins were separated by electrophoresis using a NuPAGE 4-12% Bis-Tris gradient gel in 1 x MES buffer (Life Technologies) and transferred to a Hybond-P PVDF membrane (GE Healthcare, activated in 100% (v/v) methanol) using a semi dry apparatus (Hoefer Scientific Instruments). Membranes were blocked with 7.5% (w/v) skimmed milk powder in PBST (137 mM NaCl, 2.7 mM KCl, 10 mM Na_2_HPO_4_, 1.8 mM KH_2_PO_4_ [pH7.4], 0.1% (v/v) Tween 20), washed three times in PBST, and incubated with primary antibody in 1% bovine serum albumin in PBST for 2 hours at room temperature. Following three washes in PBST, incubation with secondary antibody (Anti-rabbit horseradish peroxidase-linked antibody) was carried out in milk-PBST for 1h at room temperature, followed by three final washes in PBST. Detection of proteins was by chemiluminescence using Pierce™ ECL Western Blotting Substrate (Thermo Fisher Scientific Inc.) and ImageQuant LAS 4000 mini digital imaging system (GE Healthcare).

## Supplementary Material

Supplementary material

## Figures and Tables

**Table:** 

**Question**	**Response**
Does this article report on work with humans or animals?	Not applicable (no human or animal experimentation is reported)
Does this article include details (names, initials, hospital numbers), images, or videos relating to an individual person?	No
