# Supplementary material for: Regulation of DNA replication initiation by ParA is independent of parS location in Bacillus subtilis
Source: Microbiology (Reading). Author manuscript; Available in PMC 2023 Jul 31. (PMC7614844; doi:10.1099/mic.0.001259)
Supplement: Supplementary material [file EMS179175-supplement-Supplementary_material.pdf]

SI EXPERIMENTAL PROCEDURES

Table S1. Strains used in this study

Table S2. Plasmids used in this study

Table S3. Oligonucleotides used in this study

Table S4. Strains used in each figure

Supplementary Material
Click here to access/download;Supplementary Material;Koh_Supplementary.pdf

Regulation of DNA replication initiation by ParA is independent of *parS* location in *Bacillus subtilis*

Supplementary material

CONFIDENTIAL

Supplementary Figures

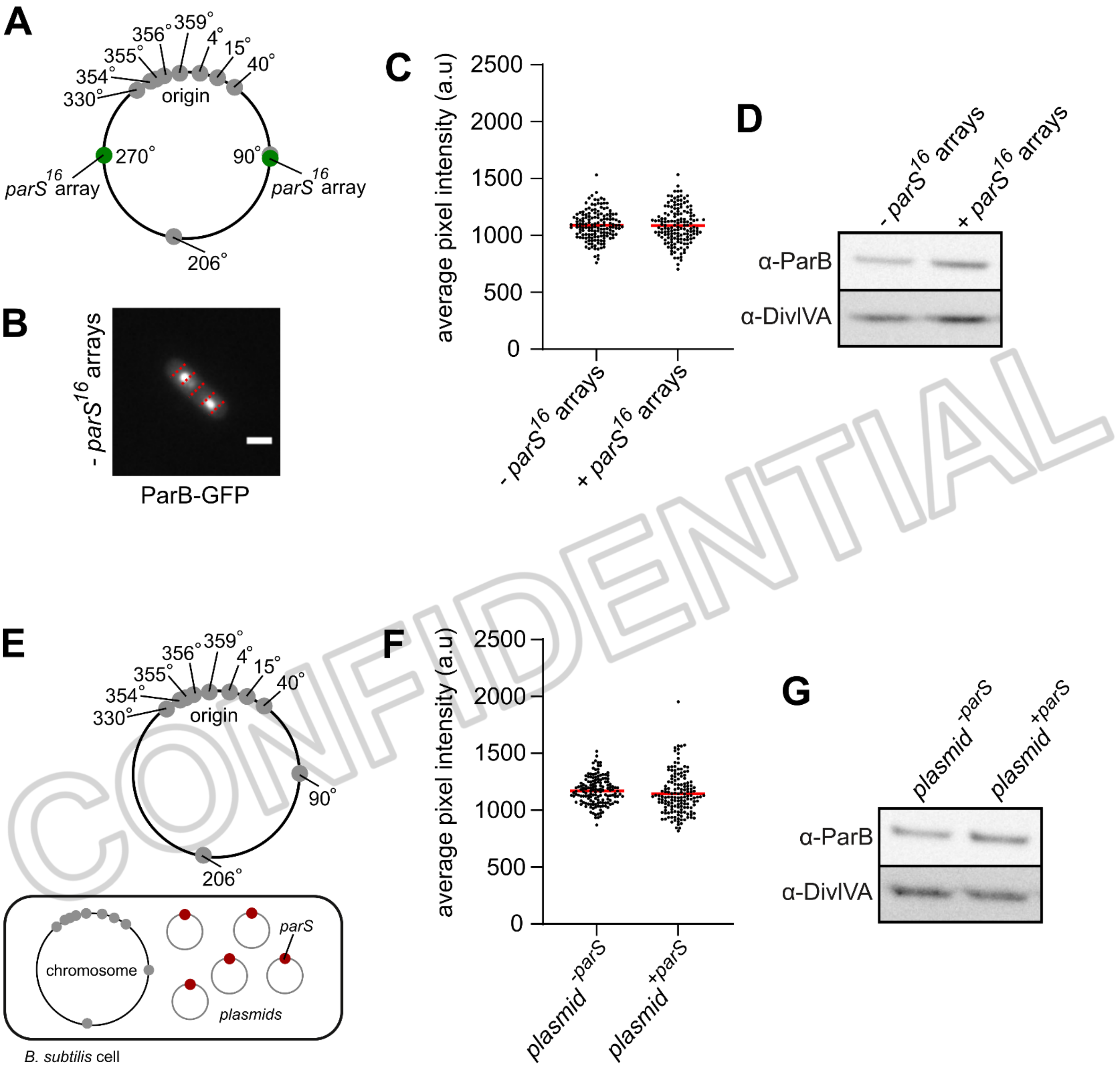

CONFIDENTIAL

Figure S1: Intracellular level of ParB remains unchanged in cells harbouring extra *parS* sites. (A) Schematic diagram showing the location of *B. subtilis* endogenous chromosomal *parS* (grey circles) and the *parS*^{16} arrays (green circles). **(B)** Fluorescent image of a representative cell used for the intensity profile plot. Dotted lines represent the area measured to generate the intensity profile of individual cells. Scale bar, 1 µm. **(C)** The average fluorescent intensity for ParB-

GFP in strains containing chromosomal *parS*[16] arrays was measured for 150 individual cells. P-value of 0.9462. **(D)** Intracellular ParB level in strains containing chromosomal *parS*[16] arrays was detected by immunoblot using an α-ParB antibody (top panel) and α-DivIVA antibody as a loading control (bottom panel). **(E)** Schematic diagram showing the location of *B. subtilis* endogenous chromosomal *parS* (grey circles) and plasmid containing a single *parS* (red circles). **(F)** The average fluorescent intensity for ParB-GFP in strains containing extrachromosomal *parS* was measured for 150 individual cells. P-value of 0.1377. **(G)** Intracellular ParB level in strains containing extrachromosomal *parS* was detected by Western blot using an α-ParB antibody (top panel) and α-DivIVA antibody as a loading control (bottom panel).

CONFIDENTIAL

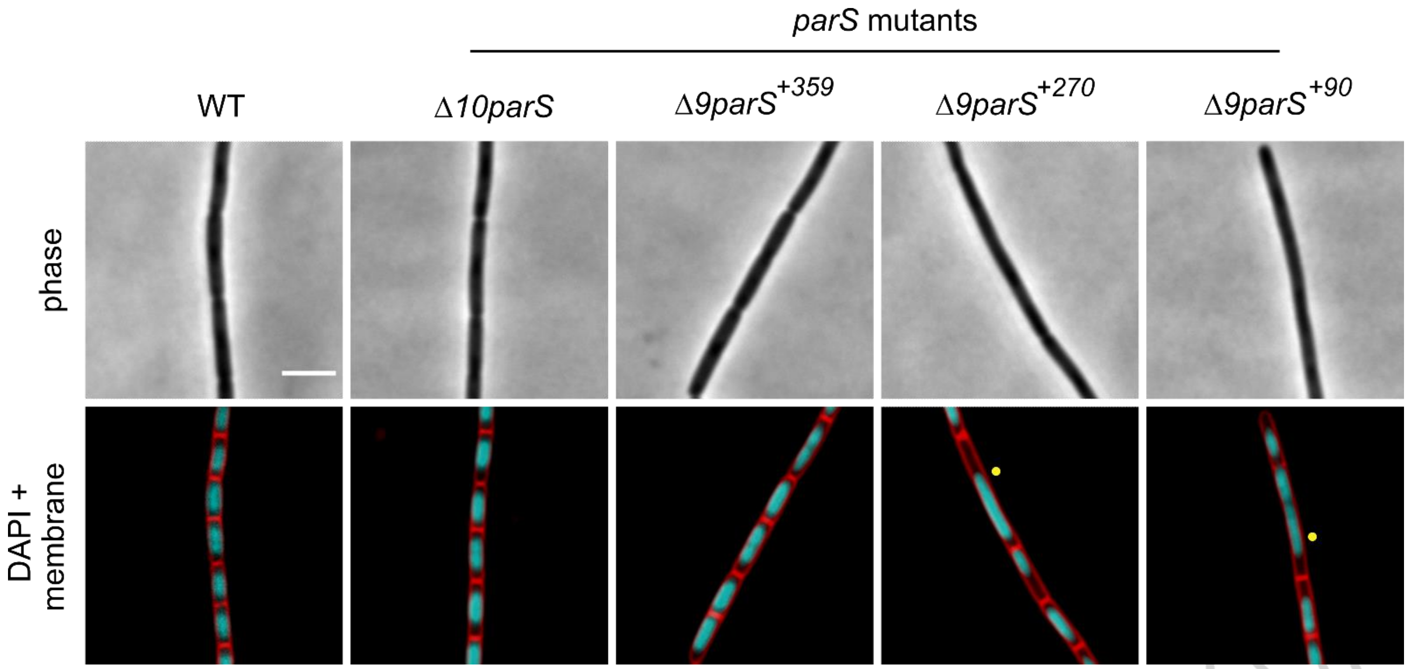

Figure S2: Chromosome organization was disrupted when *parS* was located away from the origin. DAPI staining of chromosomal DNA in strains that differ in the location of *parS*. Phase contrast (top panel) and membrane dye FM 5-95 combined with DNA stain DAPI (bottom panel). Scale bar, 3μm.

CONFIDENTIAL

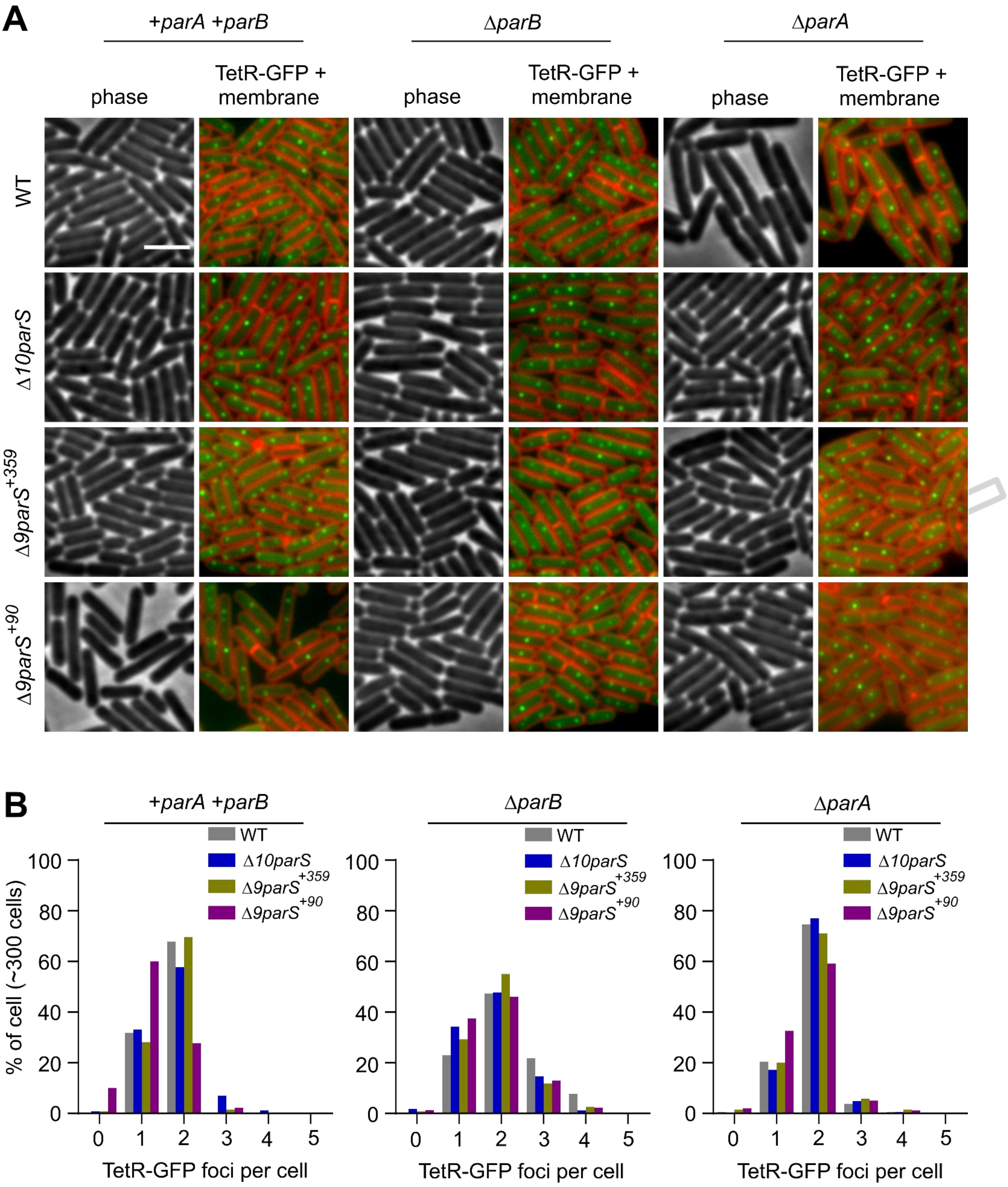

Figure S3: A single *parS* redistribute ParB to disrupt chromosome origin segregation. (A)

Origin localization in a wild-type strain and strains that differ in the location of *parS*. The *oriC*

region was labelled using an array of *tetO* operators bound by TetR-GFP. Phase contrast (left panel), and membrane dye FM 5-95 combined with TetR-GFP (right panel). **(B)** ParB dependent decrease when *parS* was located away from the origin. Scale bar, 3μm.

CONFIDENTIAL

Supplementary Table 1: Strains used in this study

Strains	Genotype[a]	Construction[b]	Source
B. subtilis 168CA	*trpC2 wild type*	-	[1]
B. subtilis 168CA AK47	*trpC2 tetR-gfp(spc)/tetO*$^{-7}$*(ery)*	-	[2]
B. subtilis 168CA AK83	*trpC2 cotS::parS*^{16} *array cat*	PSL23 → 168CA	this work
B. subtilis 168CA AK87	*trpC2 parA-parB::neo tetR-gfp/tetO*$^{-7}$ *parS*^{16} *arrays*	AK83 → AK99	this work
B. subtilis 168CA AK89	*trpC2 ΔparA::neo tetR-gfp/tetO*$^{-7}$ *parS*^{16} *arrays*	HM766 → AK97	this work
B. subtilis 168CA AK91	*trpC2 yheH::parS*^{16} *array cat*	PSL25 → 168CA	this work
B. subtilis 168CA AK93	*trpC2 yheH::parS*^{16} *array tet*	pcat::tet → AK91	this work
B. subtilis 168CA AK95	*trpC2 tetR-gfp/tetO*$^{-7}$ *yheH::parS*^{16} *array tet*	AK93 → AK47	this work
B. subtilis 168CA AK97	*trpC2 tetR-gfp/tetO*$^{-7}$ *parS*^{16} *arrays*	AK83 → AK95	this work
B. subtilis 168CA AK99	*trpC2 parA-parB::neo tetR-gfp/tetO*$^{-7}$ *yheH::parS*^{16} *array tet*	HM765 → AK95	this work
B. subtilis 168CA AK123	*trpC2 ΔparB::neo tetR-gfp/tetO*$^{-7}$ *parS*^{16} *arrays*	HM907 → AK97	this work
B. subtilis 168CA AK150	*trpC2 parS*^{16} *arrays*	AK83 → AK93	this work
B. subtilis 168CA AK153	*trpC2 parB-gfp::neo parS*^{16} *arrays*	HM756 → AK150	this work
B. subtilis PY79 AK169	*tetR-gfp/tetO*$^{-7}$	BKM865 → BKM918	this work
B. subtilis PY79 AK171	Δ*8parS tetR-gfp/tetO*$^{-7}$	AK47[c] → BNS1657	this work
B. subtilis PY79 AK177	Δ*8parS parA-parB::neo tetR-gfp/tetO*$^{-7}$	pHM142 → AK171	this work
B. subtilis PY79 AK179	Δ*8parS ΔparB::neo tetR-gfp/tetO*$^{-7}$	pHM456 → AK171	this work
B. subtilis PY79 AK181	*parA-parB::neo tetR-gfp/tetO*$^{-7}$	pHM48 → AK169	this work
B. subtilis PY79 AK183	*ΔparB::neo tetR-gfp/tetO*$^{-7}$	HM907 → AK169	this work
B. subtilis PY79 AK187	*ΔparA::neo tetR-gfp/tetO*$^{-7}$	pAK98 → AK169	this work
B. subtilis 168CA AK199	*trpC2 gfp-parA::neo parS*^{16} *arrays*	HM740 → AK150	this work
B. subtilis PY79 AK205	Δ*9parS (native parS*^{90}*)*	-	[3]
B. subtilis PY79 AK207	Δ*9parS parA-parB::neo tetR-gfp/tetO*$^{-7}$	pAK09 → AK177	this work
B. subtilis PY79 AK209	Δ*9parS ΔparB::neo tetR-gfp/tetO*$^{-7}$	pAK09 → AK179	this work
B. subtilis PY79 AK213	Δ*9parS (native parS*^{206}*)*	pAK23 → BNS1657	this work
B. subtilis PY79 AK215	Δ*10parS*	-	[3]
B. subtilis PY79 AK225	Δ*10parS parA-parB::neo tetR-gfp/tetO*$^{-7}$	pAK23 → AK207	this work
B. subtilis PY79 AK227	Δ*10parS ΔparB::neo tetR-gfp/tetO*$^{-7}$	pAK23 → AK209	this work
B. subtilis PY79 AK239	*parA-parB::neo*	pAK26 → PY79	this work
B. subtilis PY79 AK241	Δ*parB::neo*	-	[3]
B. subtilis PY79 AK243	Δ*10parS parA-parB::neo*	-	[3]
B. subtilis PY79 AK245	Δ*10parS* Δ*parB::neo*	pAK30 → AK215	this work
B. subtilis PY79 AK259	Δ*9parS*$^{+359}$ *parA-parB::neo*	pAK26 → AK215	this work
B. subtilis PY79 AK261	Δ*9parS*$^{+359}$ Δ*parB::neo*	pAK28 → AK215	this work
B. subtilis PY79 AK277	*gfp-parA::neo*	pHM23 → PY79	this work
B. subtilis PY79 AK281	Δ*10parS gfp-parA::neo*	pAK48 → AK215	this work

CONFIDENTIAL

B. subtilis PY79 AK305	*Δ9parS*$^{+359}$ *gfp-parA::neo*	pHM23 → AK215	this work
B. subtilis PY79 AK315	*Δ9parS*$^{+90}$	pAK56 → AK205	this work
B. subtilis PY79 AK323	*Δ9parS*$^{+90}$ *parA-parB::neo*	pAK32 → AK315	this work
B. subtilis PY79 AK325	*Δ9parS*$^{+90}$ *ΔparB::neo*	pAK30 → AK315	this work
B. subtilis PY79 AK367	*parB-gfp::neo*	pAK82 → PY79	this work
B. subtilis PY79 AK369	*Δ10parS parB-gfp::neo*	pAK83 → AK215	this work
B. subtilis PY79 AK373	*Δ9parS*$^{+90}$ *parB-gfp::neo*	pAK83 → AK315	this work
B. subtilis PY79 AK399	*Δ9parS*$^{+90}$ *gfp-parA::neo*	pAK48 → AK315	this work
B. subtilis PY79 AK405	*Δ9parS*$^{+90}$ *tetR-gfp/tetO*$^{-7}$	pAK56 → AK207	this work
B. subtilis PY79 AK519	*Δ9parS tetR-gfp/tetO*$^{-7}$	pAK23 → AK171	this work
B. subtilis PY79 AK521	*Δ10parS tetR-gfp/tetO*$^{-7}$	pAK09 → AK519	this work
B. subtilis 168CA AK547	*trpC2 Δspo0J gfp-parA::neo*	HM13 → 168CA	this work
B. subtilis PY79 AK553	*Δ9parS*$^{+90}$ *Δ(parA-parB)::neo*	pAK100 → AK315	this work
B. subtilis PY79 AK555	*Δ9parS*$^{+90}$ *ΔparA::neo*	pAK101 → AK315	this work
B. subtilis PY79 AK557	*ΔparA::neo*	pAK98 → PY79	this work
B. subtilis PY79 AK559	*Δ(parA-parB)::neo*	pAK99 → PY79	this work
B. subtilis PY79 AK561	*Δ9parS*$^{+359}$ *Δ(parA-parB)::neo*	pAK99 → AK215	this work
B. subtilis PY79 AK563	*Δ10parS Δ(parA-parB)::neo*	pAK100 → AK215	this work
B. subtilis 168CA AK569	*trpC2 Δspo0J gfp-soj::neo plasmid*$^{-parS}$	pHM430 → AK547	this work
B. subtilis 168CA AK571	*trpC2 Δspo0J gfp-soj::neo plasmid*$^{+parS}$	pHM432 → AK547	this work
B. subtilis 168CA AK573	*trpC2 Δ(parA-parB) tetR-gfp/tetO*$^{-7}$	HM749 → AK47	this work
B. subtilis 168CA AK575	*trpC2 Δ(parA-parB)::neo tetR-gfp/tetO*$^{-7}$ *parS*^{16} *arrays*	HM749 → AK97	this work
B. subtilis 168CA AK577	*trpC2 ΔparB gfp-parA::neo parS*^{16} *arrays*	AK547 → AK150	this work
B. subtilis 168CA AK585	*trpC2 Δsoj Δspo0J tetR-yfp/tetO*$^{-150}$	HM194 → 168CA	this work
B. subtilis 168CA AK587	*trpC2 Δsoj Δspo0J tetR-yfp/tetO*$^{-150}$ *plasmid*$^{-parS}$	pHM430 → AK585	this work
B. subtilis 168CA AK589	*trpC2 Δsoj Δspo0J tetR-yfp/tetO*$^{-150}$ *plasmid*$^{+parS}$	pHM432 → AK585	this work
B. subtilis PY79 AK591	*Δ9parS*$^{+359}$ *tetR-gfp/tetO*$^{-7}$	pAK26 → AK521	this work
B. subtilis PY79 AK593	*Δ9parS*$^{+359}$ *parB-gfp::neo*	pAK82 → AK215	this work
B. subtilis PY79 AK597	*Δ9parS*$^{+270}$ *parB-gfp::neo*	pAK180 → AK369	this work
B. subtilis PY79 AK599	*Δ9parS*$^{+270}$ *gfp-parA::neo*	pAK180 → AK281	this work
B. subtilis PY79 AK603	*Δ9parS*$^{+270}$ *soj-parB::neo*	pAK180 → AK243	this work
B. subtilis PY79 AK605	*Δ9parS*$^{+270}$ *ΔparB::neo*	pAK180 → AK245	this work
B. subtilis PY79 AK607	*Δ9parS*$^{+270}$ *Δ(parA-parB)::neo*	pAK180 → AK563	this work
B. subtilis PY79 AK615	*Δ9parS*$^{+90}$ *ΔparB::neo tetR-gfp/tetO*$^{-7}$	pAK56 → AK209	this work
B. subtilis PY79 AK617	*Δ10parS ΔparA::neo*	pAK101 → AK215	this work
B. subtilis PY79 AK639	*Δ9parS*$^{+359}$ *ΔparB::neo tetR-gfp/tetO*$^{-7}$	pAK28 → AK521	this work
B. subtilis PY79 AK649	*Δ9parS*$^{+270}$ *ΔparA::neo*	pAK180 → AK617	this work
B. subtilis PY79 AK667	*Δ8parS*$^{+359}$ *ΔparA::neo*	pHM160 → AK205	this work
B. subtilis PY79 AK669	*Δ9parS*$^{+359}$ *ΔparA::neo*	AK213 → AK667	this work

CONFIDENTIAL

B. subtilis PY79 AK673	*Δ9parS*$^{+90}$ *ΔparA::neo tetR-gfp*	AK171 → AK555	this work
B. subtilis PY79 AK675	*Δ10parS ΔparA::neo tetR-gfp*	AK171 → AK617	this work
B. subtilis PY79 AK677	*Δ9parS*$^{+359}$ *ΔparA::neo tetR-gfp*	AK171 → AK669	this work
B. subtilis PY79 AK683	*Δ9parS*$^{+90}$ *ΔparA::neo tetR-gfp/tetO*$^{-7}$	AK171 → AK673	this work
B. subtilis PY79 AK685	*Δ10parS ΔparA::neo tetR-gfp/tetO*$^{-7}$	AK171 → AK675	this work
B. subtilis PY79 AK687	*Δ9parS*$^{+359}$ *ΔparA::neo tetR-gfp/tetO*$^{-7}$	AK171 → AK677	this work
B. subtilis PY79 BKM865	*tetR-gfp/tetO*$^{-7}$*::cat*	-	[4]
B. subtilis PY79 BKM918	*tetO*$^{-7}$*::ery*	-	[4]
B. subtilis PY79 BNS1657	*Δ8parS*	-	[5]
B. subtilis 168ED HM4	*trpC2 gfp-parA::neo*	-	[6]
B. subtilis 168ED HM13	*trpC2 ΔparB gfp-parA::neo*	-	[6]
B. subtilis 168ED HM34	*trpC2 parA-parB::neo*	-	[6]
B. subtilis 168ED HM61	*trpC2 parB-gfp::neo*	-	[7]
B. subtilis 168ED HM130	*trpC2 (spo0J tetO*$^{-150}$*):: neo cgeD::(*P_{pen}*-tetR-yfp) tet*	-	[6]
B. subtilis 168ED HM168	*trpC2 (Δspo0J tetO*$^{-150}$*)::neo cgeD::(*P_{pen}*-tetR-yfp) tet*	-	[6]
B. subtilis 168ED HM171	*trpC2 (Δsoj tetO*$^{-150}$*)::neo cgeD::(*P_{pen}*-tetR-yfp) tet*	-	[6]
B. subtilis 168ED HM183	*trpC2 Δ(parA-parB)::neo*	-	[6]
B. subtilis 168ED HM194	*trpC2 (Δsoj Δspo0J tetO*$^{-150}$*)::neo cgeD::(*P_{pen}*-tetR-yfp) tet*	-	[6]
B. subtilis 168ED HM226	*trpC2 pheA ΔparB::neo*	-	[6]
B. subtilis 168ED HM227	*trpC2 pheA ΔparA::neo*	-	[6]
B. subtilis 168CA HM740	*trpC2 gfp-parA::neo*	HM4 → 168CA	this work
B. subtilis 168CA HM747	*trpC2 parA-parB::neo*	HM34 → 168CA	this work
B. subtilis 168CA HM748	*trpC2 ΔparA::neo*	HM227 → 168CA	this work
B. subtilis 168CA HM749	*trpC2 Δ(parA-parB)::neo*	HM183 → 168CA	this work
B. subtilis 168CA HM754	*trpC2 ΔparB::neo*	HM226 → 168CA	this work
B. subtilis 168CA HM756	*trpC2 parB-gfp::neo*	HM61 → 168CA	this work
B. subtilis 168CA HM765	*trpC2 parA-parB::neo tetR-gfp/tetO*$^{-7}$	HM747 → AK47	this work
B. subtilis 168CA HM766	*trpC2 ΔparA::neo tetR-gfp/tetO*$^{-7}$	HM748 → AK47	this work
B. subtilis 168CA HM821	*trpC2 spo0J-gfp::neo plasmid*$^{-parS}$	pHM430 → HM756	this work
B. subtilis 168CA HM823	*trpC2 spo0J-gfp::neo plasmid*$^{+parS}$	pHM432 → HM756	this work
B. subtilis 168CA HM831	*trpC2 Δspo0J tetR-yfp/tetO*$^{-150}$	HM168 → 168CA	this work
B. subtilis 168CA HM832	*trpC2 Δsoj tetR-yfp/tetO*$^{-150}$	HM171 → 168CA	this work
B. subtilis 168CA HM834	*trpC2 Δspo0J tetR-yfp/tetO*$^{-150}$ *plasmid*$^{-parS}$	pHM430 → HM831	this work
B. subtilis 168CA HM835	*trpC2 Δsoj tetR-yfp/tetO*$^{-150}$ *plasmid*$^{-parS}$	pHM430 → HM832	this work
B. subtilis 168CA HM837	*trpC2 Δspo0J tetR-yfp/tetO*$^{-150}$ *plasmid*$^{+parS}$	pHM432 → HM831	this work
B. subtilis 168CA HM838	*trpC2 Δsoj tetR-yfp/tetO*$^{-150}$ *plasmid*$^{+parS}$	pHM432 → HM832	this work
B. subtilis 168CA HM853	*trpC2 tetR-yfp/tetO*$^{-150}$	HM130 → 168CA	this work
B. subtilis 168CA HM855	*trpC2 tetR-yfp/tetO*$^{-150}$ *plasmid*$^{-parS}$	pHM430 → HM853	this work

CONFIDENTIAL

B. subtilis 168CA HM856	*trpC2 tetR-yfp/tetO^{-150} plasmid^{+parS}*	pHM432 → HM853	this work
B. subtilis 168CA HM870	*trpC2 gfp-soj::neo plasmid^{-parS}*	pHM430 → HM740	this work
B. subtilis 168CA HM871	*trpC2 gfp-soj::neo plasmid^{+parS}*	pHM432 → HM740	this work
B. subtilis 168CA HM907	*trpC2 ΔparB::neo tetR-gfp/tetO^{-7}*	HM754 → AK47	this work
B. subtilis JH642 PSL23	*trpC2 Δ6parS parB-gfp cotS::parS^{16} array cat*	-	[8]
B. subtilis JH642 PSL25	*trpC2 Δ6parS parB-gfp yheH::parS^{16} array cat*	-	[8]
B. subtilis PY79	wild type prototroph	-	[9]

[a] *B. subtilis* antibiotic resistance markers are listed respectively. *neo*, kanamycin resistance; *ery*, erythromycin resistance; *cat*, chloramphenicol resistance; *spc*, spectinomycin resistance; *tet*, tetracycline resistance.

[b] Plasmids or genomic DNA of the indicated strain transformed into the parent strain used in the genetic construction.

[c] DNA fragments amplified from AK47 gDNA using primer pairs oAK5/oAK6 and oAK7/oAK8.

CONFIDENTIAL

Supplementary Table 2: Plasmids used in this study

Plasmid	Genotype[a]	Construction[b]	Source
pAK09	*bla cat 'sigX ΔparS*^{206}*::zeo resE'*	-	[3]
pAK23	*bla cat 'yhaX ΔparS*^{90}*::tet hemZ'*	-	[3]
pAK26	*bla cat parB::neo*	-	[3]
pAK28	*bla cat ΔparB::neo*	-	[3]
pAK30	*bla cat ΔparB ΔparS*^{359}*::neo*	pHM456 (oAK55/oGJS431) → pHM23	this work
pAK32	*bla cat parB ΔparS*^{359}*::neo*	-	[3]
pAK48	*bla cat gfp-parA ΔparS*^{359}*::neo*	pHM142 (oAK52/oAK53) → pHM23	this work
pAK56	*bla cat 'yhaX parS*^{90}*::tet hemZ'*	pHM48 (oAK61/oAK62) → pAK23	this work
pAK82[c]	*bla cat parB-gfp::neo*	pHM110 → pAK26	this work
pAK83[c]	*bla cat parB-gfp ΔparS*^{359}*::neo*	pHM110 → pAK32	this work
pAK98	*bla cat ΔparA::neo*	pHM160 (oAK58/oHM20) → pAK26	this work
pAK99	*bla cat Δ(parA-parB)::neo*	pHM160 (oAK58/oHM20) → pAK28	this work
pAK100	*bla cat Δ(parA-parB ΔparS*^{359}*)::neo*	pHM160 (oAK58/oHM20) → pAK30	this work
pAK101	*bla cat ΔparA ΔparS*^{359}*::neo*	pHM160 (oAK58/oHM20) → pAK32	this work
pAK128	*bla cat 'ytcC::zeo*	160CA (oAK191/oAK192) → pHM457	this work
pAK130	*bla cat zeo ytxO'*	160CA (oAK189/oAK190) → pHM457	this work
pAK136[c]	*bla cat 'ytcC::zeo ytxO'*	pAK130 → pAK128	this work
pAK178	*bla cat 'ytcC::erm ytxO'*	pHM432 (oAK227/oAK257) → pAK136	this work
pAK180	*bla cat 'ytcC::erm parS*^{270} *ytxO'*	pHM48 (oAK216/oAK217) → pAK178	this work
pcat::tet	*bla cat::tet*	-	[10]
pHM23	*bla cat gfp-parA::neo*	-	[6]
pHM48	*bla cat parB::neo*	-	[6]
pHM52	*bla cat ΔparB::neo*	-	[6]
pHM59	*bla cat Δ(parA-parB)::tet*	-	[6]
pHM110	*bla cat parB-gfp::neo*	-	[7]
pHM142	*bla cat parB ΔparS*^{359}*::neo*	-	[3]
pHM160	*bla cat ΔparA::neo*	-	[6]
pHM430	*ery*	pNZ8907	[11]
pHM432	*ery 'spo0J-parS-spo0J'*	168CA (oAK61/oAK62) → pHM430	this work
pHM456[c]	*bla cat ΔparB ΔparS*^{359}*::neo*	pHM142 → pHM52	this work
pHM457	*bla cat zeo*	-	[3]

[a] *E. coli* and *B. subtilis* antibiotic resistance markers are listed first and second respectively. *bla,* ampicillin resistance; *kan*, kanamycin resistance; *ery*, erythromycin resistance; *cat*, chloramphenicol resistance; *spc*, spectinomycin resistance; *tet*, tetracycline resistance; *phl*, phleomycin resistance; zeomycin resistance; *zeo*.

[b] DNA fragments amplified with primer pair indicated in parentheses using template from either plasmids or genomic DNA indicated in parentheses before the amplified products are cloned into the parent plasmid used in the genetic construction.

[c] DNA fragments are digested by restriction enzymes and cloned into the parent plasmid used in the genetic construction.

CONFIDENTIAL

Supplementary Table 3: Oligonucleotides used in this study

Primer	Sequence (5'→3')
oAK52	AAGGGAAGGGCCGCGGTCAAGAGGTGCGGAAGTATATTTAG
oAK53	AAGGGAAGGGTCTAGAACTAGTGGATCCCCCGGGCTGC
oAK55	AATTTAATTTCCTAGGTAAGGAAAAATCATAGCAATTACGAAC
oAK58	TTTATGCTTCCGGCTCGTATG
oAK61	AATTTAATTTGCGGCCGCGACTGCTGACACTGCCAG
oAK62	AATTTAATTTGCGGCCGCCATTTATGATTCTCGTTCAGAC
oAK91	AATTTAATTTGAGCTCGCGTTCTCTAATTTCACAAGAGG
oAK92	GATTGGAAATACGGATTCTG
oAK189	AAGGGAATTCTCGAGGTGACTTTATGCCTTGCGCTTTAC
oAK190	AAGGGAATTGGTACCGCCTCTATTTCTAAACTGGATCTCGAC
oAK216	AAGGGAATTTCTAGAGACTGCTGACACTGCC
oAK217	AAGGGAATTTCTAGACATTTATGATTCTCGTTCAGAC
oAK227	AAGGGAATTGGATCCGACTCATAGAATTATTTCCTCCCG
oAK257	AATTTAATTTCTCGAGTATCACGAGGCCCTTTCGTCTTCAAGAATTGATCC
oGJS431	GGTGGTCCCGCGGCATCTTCGTTTGAAAGATGC GGC
oHM20	TTTTCTTGGCTGATAAGGATTAGGGCGTAAATCG

CONFIDENTIAL

Supplementary Table 4: Strains used in each figures

Fig.1	HM756 (*parB-gfp*), AK153 (*parB-gfp parS*^{16} arrays)
Fig. 2	HM821 (*parB-gfp plasmid*$^{-parS}$), HM823 (*parB-gfp plasmid*$^{+parS}$)
Fig. 3	HM765 (*tetR-gfp/tetO*$^{-7}$), AK87 (*tetR-gfp/tetO*$^{-7}$ *parS*^{16} arrays), HM907 (*tetR-gfp/tetO*$^{-7}$Δ*parB*), AK123 (*tetR-gfp/tetO*$^{-7}$ *parS*^{16} arrays Δ*parB*), HM766 (*tetR-gfp/tetO*$^{-7}$ Δ*parA*), AK89 (*tetR-gfp/tetO*$^{-7}$ *parS*^{16} arrays Δ*parA*)
Fig. 4	HM855 (*tetR-yfp/tetO*$^{-7}$ *plasmid*$^{-parS}$), HM856 (*tetR-yfp/tetO*$^{-7}$ *plasmid*$^{+parS}$), HM834 (Δ*parB tetR-yfp/tetO*$^{-7}$ *plasmid*$^{-parS}$), HM837 (Δ*parB tetR-yfp/tetO*$^{-7}$ *plasmid*$^{+parS}$), HM835 (Δ*parA tetR-yfp/tetO*$^{-7}$ *plasmid*$^{-parS}$), HM838 (Δ*parA tetR-yfp/tetO*$^{-7}$ *plasmid*$^{+parS}$)
Fig. 5	HM765 (*tetR-gfp/tetO*$^{-7}$), AK87 (*tetR-gfp/tetO*$^{-7}$ *parS*^{16} arrays), HM907 (*tetR-gfp/tetO*$^{-7}$*ΔparB*), AK123 (*tetR-gfp/tetO*$^{-7}$ *parS*^{16} arrays *ΔparB*), AK573 (*tetR-gfp/tetO*$^{-7}$ *ΔparA ΔparB*), AK575 (*tetR-gfp/tetO*$^{-7}$ *parS*^{16} arrays *ΔparA ΔparB*), HM766 (*tetR-gfp/tetO*$^{-7}$ *ΔparA*), AK89 (*tetR-gfp/tetO*$^{-7}$ *parS*^{16} arrays *ΔparA*), HM740 (*gfp-parA*), AK199 (*gfp-parA parS*^{16} arrays), AK547 (*gfp-parA ΔparB*), AK577 (*gfp-parA ΔparB parS*^{16} arrays), HM855 (*tetR-yfp/tetO*$^{-7}$ *plasmid*$^{-parS}$), HM856 (*tetR-yfp/tetO*$^{-7}$ *plasmid*$^{+parS}$), HM834 (Δ*spo0J tetR-yfp/tetO*$^{-7}$ *plasmid*$^{-parS}$), HM837 (Δ*spo0J tetR-yfp/tetO*$^{-7}$ *plasmid*$^{+parS}$), AK587 (Δ*soj* Δ*spo0J tetR-yfp/tetO*$^{-7}$ *plasmid*$^{-parS}$), AK589 (Δ*soj* Δ*spo0J tetR-yfp/tetO*$^{-7}$ *plasmid*$^{+parS}$), HM835 (Δ*soj tetR-yfp/tetO*$^{-7}$ *plasmid*$^{-parS}$), HM838 (Δ*soj tetR-yfp/tetO*$^{-7}$ *plasmid*$^{+parS}$), HM870 (*gfp-soj plasmid*$^{-parS}$), HM871 (*gfp-soj plasmid*$^{+parS}$), AK569 (*Δspo0J gfp-soj plasmid*$^{-parS}$), AK571 (*Δspo0J gfp-soj plasmid*$^{+parS}$).
Fig. 6	AK367 (*parB-gfp*), AK369 (*parB-gfp* Δ10*parS*), AK593 (*parB-gfp* Δ9*parS*$^{+359}$), AK597 (*parB-gfp* Δ9*parS*$^{+270}$), AK373 (*parB-gfp* Δ9*parS*$^{+90}$), AK239 (wild type), AK243 (Δ10*parS*), AK259 (Δ9*parS*$^{+359}$), AK603 (Δ9*parS*$^{+270}$), AK323 (Δ9*parS*$^{+90}$), AK241 (Δ*parB*), AK245 (Δ*parB* Δ10*parS*), AK261 (Δ*parB* Δ9*parS*$^{+359}$), AK605 (Δ*parB* Δ9*parS*$^{+270}$), AK325 (Δ*parB* Δ9*parS*$^{+90}$), AK559 (Δ*parA* Δ*parB*), AK563 (Δ*parA* Δ*parB* Δ10*parS*), AK561 (Δ*parA* Δ*parB* Δ9*parS*$^{+359}$), AK607 (Δ*parA* Δ*parB* Δ9*parS*$^{+270}$), AK553 (Δ*parA* Δ*parB* Δ9*parS*$^{+90}$), AK557 (Δ*parA*), AK617 (Δ*parA* Δ10*parS*), AK669 (Δ*parA* Δ9*parS*$^{+359}$), AK649 (Δ*parA* Δ9*parS*$^{+270}$), AK555 (Δ*parA* Δ9*parS*$^{+90}$), AK277 (*gfp-parA*), AK281 (*gfp-parA* Δ10*parS*), AK305 (*gfp-parA* Δ9*parS*$^{+359}$), AK599 (*gfp-parA* Δ9*parS*$^{+270}$), AK399 (*gfp-parA* Δ9*parS*$^{+90}$)

CONFIDENTIAL

Fig. S1 HM756 (*parB-gfp*), AK153 (*parB-gfp parS*^{16} arrays), HM821 (*parB-gfp plasmid*$^{-parS}$), HM823 (*parB-gfp plasmid*$^{+parS}$), HM765 (*tetR-gfp/tetO*$^{-7}$), AK87 (*tetR-gfp/tetO*$^{-7}$ *parS*^{16} arrays), HM855 (*tetR-yfp/tetO*$^{-7}$ *plasmid*$^{-parS}$), HM856 (*tetR-yfp/tetO*$^{-7}$ *plasmid*$^{+parS}$)

Fig. S2 AK239 (wild type), AK243 (Δ10*parS*), AK259 (Δ9*parS*$^{359+}$), AK603 (Δ9*parS*$^{270+}$), AK323 (Δ9*parS*$^{90+}$)

Fig. S3 AK181 (*tetR-gfp/tetO*$^{-7}$), AK225 (*tetR-gfp/tetO*$^{-7}$ Δ10*parS*), AK591 (*tetR-gfp/tetO*$^{-7}$ Δ9*parS*$^{359+}$), AK405 (*tetR-gfp/tetO*$^{-7}$ Δ9*parS*$^{90+}$), AK183 (*tetR-gfp/tetO*$^{-7}$ Δ*parB*), AK227 (*tetR-gfp/tetO*$^{-7}$ Δ*parB* Δ10*parS*), AK639 (*tetR-gfp/tetO*$^{-7}$ Δ*parB* Δ9*parS*$^{359+}$), AK615 (*tetR-gfp/tetO*$^{-7}$ Δ*parB* Δ9*parS*$^{90+}$), AK187 (*tetR-gfp/tetO*$^{-7}$ Δ*parA*), AK685 (*tetR-gfp/tetO*$^{-7}$ Δ*parA* Δ10*parS*), AK687 (*tetR-gfp/tetO*$^{-7}$ Δ*parA* Δ9*parS*$^{359+}$), AK683 (*tetR-gfp/tetO*$^{-7}$ Δ*parA* Δ9*parS*$^{90+}$)

CONFIDENTIAL

References

1. **Kunst F, Ogasawara N, Moszer I, Albertini AM, Alloni G et al.** The complete genome sequence of the Gram-positive bacterium Bacillus subtilis. *Nature* 1997;390(6657):249-256.
2. **Murray H, Koh A**. Multiple Regulatory Systems Coordinate DNA Replication with Cell Growth in Bacillus subtilis. *PLoS Genet* 2014;10(10):e1004731.
3. **Marbouty M, Le Gall A, Cattoni DI, Cournac A, Koh A et al.** Condensin- and Replication-Mediated Bacterial Chromosome Folding and Origin Condensation Revealed by Hi-C and Super-resolution Imaging. *Mol Cell* 2015;59(4):588-602.
4. **Wagner JK, Marquis KA, Rudner DZ**. SirA enforces diploidy by inhibiting the replication initiator DnaA during spore formation in Bacillus subtilis. *Mol Microbiol* 2009;73(5):963-974.
5. **Sullivan NL, Marquis KA, Rudner DZ**. Recruitment of SMC by ParB-parS Organizes the Origin Region and Promotes Efficient Chromosome Segregation. *Cell* 2009;137(4):697-707.
6. **Murray H, Errington J**. Dynamic Control of the DNA Replication Initiation Protein DnaA by Soj/ParA. *Cell* 2008;135(1):74-84.
7. **Leaver M, Dominguez-Cuevas P, Coxhead JM, Daniel RA, Errington J**. Life without a wall or division machine in Bacillus subtilis. *Nature* 2009;457(7231):849-853.
8. **Lee PS, Lin DCH, Moriya S, Grossman AD**. Effects of the chromosome partitioning protein Spo0J (ParB) on oriC positioning and replication initiation in Bacillus subtilis. *Journal of Bacteriology* 2003;185(4):1326-1337.
9. **Youngman PJ, Perkins JB, Losick R**. Genetic Transposition and Insertional Mutagenesis in Bacillus-Subtilis with Streptococcus-Faecalis Transposon Tn917. *Proceedings of the National Academy of Sciences of the United States of America-Biological Sciences* 1983;80(8):2305-2309.
10. **Steinmetz M, Richter R**. Plasmids Designed to Alter the Antibiotic-Resistance Expressed by Insertion Mutations in Bacillus-Subtilis, through in-Vivo Recombination. *Gene* 1994;142(1):79-83.
11. **Bongers RS, Veening JW, Van Wieringen M, Kuipers OP, Kleerebezem M**. Development and characterization of a subtilin-regulated expression system in Bacillus subtilis: strict control of gene expression by addition of subtilin. *Appl Environ Microbiol* 2005;71(12):8818-8824.

CONFIDENTIAL
